# Overweight-Related Hypertension in Middle-Aged Men Is Linked to Elevated Leptin, TNF-α, IL-6, Cholesterol, and Reduced Testosterone

**DOI:** 10.3390/pathophysiology32010007

**Published:** 2025-02-02

**Authors:** Shalan Alaamri, Abdulhalim S. Serafi, Zahir Hussain, Shouq K. Bafail, Mohammed A. Bafail, Lusine Demirkhanyan, Christopher S. Gondi, Sumera Sohail

**Affiliations:** 1 Department of Medicine, College of Medicine, University of Jeddah, Jeddah 21589, Saudi Arabia; shalaamri@uj.edu.sa; 2 Department of Physiology, Faculty of Medicine, Umm Al-Qura University, Makkah 21955, Saudi Arabia; mabafail@uqu.edu.sa; 3 Department of Biology, College of Sciences, Umm Al-Qura University, Aljamoum 22254, Saudi Arabia; shouq.bafail@gmail.com; 4 Department of Internal Medicine, University of Illinois College of Medicine Peoria, Peoria, IL 61605, USA; lusinhd@uic.edu (L.D.); gondi@uic.edu (C.S.G.); 5 Department of Surgery, University of Illinois College of Medicine Peoria, Peoria, IL 61605, USA; 6 Department of Health Science Education and Pathology, University of Illinois College of Medicine Peoria, Peoria, IL 61605, USA; 7 The Grainger College of Engineering, University of Illinois at Urbana-Champaign, Urbana, IL 61801, USA; 8 Department of Physiology, University of Karachi, Karachi 75270, Pakistan; sumera_sohail@yahoo.com

**Keywords:** middle-aged men, hypertension, overweight-related hypertension, serum leptin, TNF-α, IL-6, total cholesterol, testosterone, pathophysiology

## Abstract

Background/Objectives: One of the major causes of hypertension (HT) is the transition of normal weight (NW) status to overweight (OW) status and obesity in a population, which leads to cardiovascular disease (CVD) and other disorders. A variety of factors/variables are involved in the development of HT and OW-related hypertension (OHT). However, we planned to investigate the pathophysiological role of serum leptin (Lep), tumor necrosis factor-alpha (TNF-α), interleukin-6 (IL-6), total cholesterol (TC) and serum testosterone (ST) in OHT in middle-aged men. Methods: We consulted three groups of middle-aged men (age: 51–60 years)—an HT group (n: 97, high normal weight (HNW), body mass index (BMI): 23–24.9 kg/m^2^); an OHT group (n: 97, high overweight (HOW), BMI: 28–29.9 kg/m^2^) and a normal control group (NC, n: 98, HNW)—to investigate the variations in and correlations of Lep, IL-6, TNF-α, ST, TC and other variables. Results: Significant variations were obtained for the comparisons of TNF-α, Lep, ST and TC for the patient groups. OHT vs. NC showed a significant difference for ST. OHT vs. NC and OHT vs. HT had significant variations for IL-6. Significant changes were obtained for the serum levels of TNF-α, Lep, IL-6, ST and TC among groups. Significant and positive linear associations were obtained for TNF-α, Lep, TC and IL-6. Significant and negative linear associations were found for ST plotted against Lep, TNF-α and IL-6. Conclusions: The current report provides pathophysiological evidence of the interactive role of serum Lep, TNF-α, ST, TC and IL-6 in middle-aged men with HT and OHT. We suggest that the changes we noted in the present study would be helpful for further BMI-based studies in various subcategories of NW, OW and obese subjects with/without HT.

## 1. Introduction

The age- and sex-specific data on the worldwide prevalence of hypertension (HT) show that HT is an important health challenge that must be screened, prevented and treated with high priority [1]. One of the major causes of HT is the transition of normal weight (NW) status to overweight (OW) status and obesity in a population [2], which leads to cardiovascular disease (CVD) and other disorders. It was revealed that there is a significant association between body weight (BW) and HT [3]. It was found that a high body mass index (BMI) (in either OW or obese subjects) is one of the main factors leading to several chronic disorders, e.g., CVD, diabetes mellitus (DM) and HT [4]. Furthermore, both NW and OW subjects showed an association between blood pressure (BP) and BMI [5]. OW status increases the risk of the development of high BP. The Framingham Study revealed that high BP or HT [6] and OW [7] are independent risk factors for CVD [8]. It is documented that the occurrence of HT could increase BMI [9]. The data of all age groups showed that 60–77% of obese subjects had HT with an increasing BMI, and this was significantly higher than the 34% in NW subjects [10].

The tendency to show a gradual increase in BP with an increase in BW or BMI was also noted in the general population [11] and over the course of life, including late in life [12]. The various BP range levels have been classified into three grades of HT based on systolic blood pressure (SBP)/diastolic blood pressure (DBP) in mmHg as follows: grade 1: 140–159/90–99; grade 2: 160–179/100–109; and grade 3: ≥180/≥110. However, management with a proper lifestyle and medication is essentially required regardless of any grade [13].

BP is found to be higher in OW/obese people than in non-obese people, and lifestyle changes decrease BP in OW and obese men [14]. In view of the interactive effects and association of BW (OW/obesity) and HT, combined management for both risk factors is highly recommended, even in non-hypertensive subjects [15]. Furthermore, patients with HT and OW/obesity require proper management including lifestyle changes, especially reducing BW, which decreases the risk of HT for those who have not been diagnosed as being OW or obese [16].

There are various studies documenting the association of serum leptin (Lep) with other variables including serum tumor necrosis factor-alpha (TNF-α), total cholesterol (TC), serum testosterone (ST) and interleukin-6 (IL-6) in HT [17,18,19,20] and OW-related HT (OHT) [21,22,23]. However, there are reports that contradict these findings [24,25]. We thoroughly surveyed the literature related to OW status and HT and found quite convincing evidence that OW and obesity play a significant role in the occurrence and progression of HT and vice versa [3,4,5]. However, further unraveling the aspects associated with the pathophysiology of HT under the influence of BW is required since studies conducted in the past show differing criteria for and approaches to studying OHT [4,5]. Several groups of researchers carried out studies on this topic with different perspectives [3,4,5,14,15,22]. However, a number of factors have not been studied in coherence. In such a situation, it is hard to prove the clear impact of various biochemical parameters on OHT, and it is equally difficult to explain the theoretical correlation between the biochemical parameters analyzed and HT. It was not easy for us to deal with a large number of biochemical variables in our present study.

Furthermore, it was not feasible to study other related subject groups—low overweight (LOW)-related HT (LOHT) and medium overweight (MOW)-related HT (MOHT)—and compare them with low normal weight (LNW)-related HT (LNHT) and medium normal weight (MNW)-related HT (MNHT), mainly due to funding limitations. Despite all these hurdles, based on the information obtained from our pilot studies and existing related reports, we theoretically hypothesized that some of the important biochemical parameters including serum Lep, TNF-α, IL-6, TC and ST are promising risk factors in OHT/high OHT (HOHT) compared to NHT/high NHT (HNHT) [4,18,19,20,21,22,23].

One important aspect of our hypothesis necessitates having highly well-controlled data. For that purpose, we planned to include only (a) normal controls (NCs), HT and OHT middle-aged men; (b) high OW (HOW) and HOHT men compared to high NW (HNW) and HNHT middle-aged control men; and (c) middle-aged men with grade 1 HT. During the development of the mentioned hypothesis, the studies related to the effect of weight loss and the influence of weight loss programs/therapeutic approaches and weight-decreasing factors [14,15,24,25] on Lep, TNF-α, IL-6, TC and ST in OHT provided us with potential information.

A clearly known interactive role of the mentioned factors is not yet known, which requires further studies to be conducted to explore the role of serum Lep, TNF-α, IL-6, ST, TC, fasting blood glucose (FBG) and hepcidin (Hp) in NC, HT and OHT subjects. Therefore, the purpose of the present research work was to study and investigate the involvement of the mentioned factors in HT and OHT.

## 2. Materials and Methods

### 2.1. Study Design, Participants and Data Collection

The design and detailed proposal for the present study were prepared and submitted to the Ethical Approval Committee of the Faculty of Medicine, Umm Al-Qura University. With the issuance of the ethical approval (Approval No. HAPO-02-K-012-2021-02-124), the research work was commenced. Following informed consent, participants were informed of study procedures, that the data of subjective information and the blood samples would be collected, and other tests would be carried out, during their first visit. After the selection of each subject for the present study, a detailed interview and assessment were conducted. For this purpose, proper diagnostic procedures were employed, and subjects/patients were characterized for the current study.

The present study comprised participants classified into three groups (each with an age range of 51–60 years) as NC (n: 98), HT (n: 97) and OHT (n: 97) subjects. The NC subjects were normotensive (NT) male subjects with NW and a BMI in the range of 23–24.9 kg/m^2^. The HT subjects were hypertensive male subjects with NW and a BMI in the range of 23–24.9 kg/m^2^. The OHT subjects were hypertensive male subjects with OW status and a BMI in the range of 28–29.9 kg/m^2^.

The NC and HT subjects had an HNW-related BMI, and the OHT subjects had an HOW-related BMI. Hence, the present study included subjects with HNW- and HOW-corresponding BMIs. Subjects having lower and higher levels of BMI than those mentioned for NC, HT and OHT were not included in the present study. That is, subjects having LNW- and MNW-related BMI and LOW- and MOW-related BMI were not included in the present study. Furthermore, the NT subjects had SBP/DBP of <120/<80 mmHg, and the HT subjects had SBP/DBP in the range of 140–159/90–99 (grade 1 HT) [13]. Subjects with lower and higher SBP/DBP for HT and OHT or subjects having grade 2 hypertension (SBP/DBP: 160–179/100–109) or grade 3 hypertension (SBP/DBP: ≥180/≥110) [13] were excluded from the current study.

The chronic conditions (e.g., rheumatological diseases) that could influence inflammatory parameters analyzed were exclusion criteria. All subjects in the current study were non-smokers, and they had no endocrine or reproductive disorders. They had no hypogonadism-related symptoms. The subjects had no metabolic or endocrinological disorders leading to HT and OW-related disorders like acromegaly and Cushing disease. History and comprehensive physical and clinical/laboratory examinations were performed, and it was confirmed that the subjects in the present study had no chronic kidney disease, diabetes mellitus, vision loss, coronary artery disease, stroke, gastrointestinal disturbances, osteoporosis, obesity (generalized or abdominal/central), depression, anxiety, immune suppression, asthma, rheumatoid arthritis, metabolic syndrome, epileptic seizures, sleep apnea, insomnia, other sleep disturbances, hyperthyroidism, primary aldosteronism, hyperparathyroidism or other endocrine conditions. Furthermore, we did not include subjects showing the symptoms of hypogonadism or any other disorders associated with the decreased levels of ST. Despite that, we assayed the levels of sex-hormone-binding globulin (SHBG) using an ELISA kit to confirm that age-based levels of SHBG are in the normal range for selecting the subjects for the present study and excluding subjects with hypogonadism and other disorders.

A sample size calculator was used for assessing the required number of samples for the present study. The general features of the subjects and the history of patients were obtained and recorded in a questionnaire. The present study was conducted in the Umm Al-Qura University-affiliated hospitals in Makkah, Saudi Arabia, from June 2021 to December 2022. Considering the previous report [26], it was planned to collect the data for serum Lep, TNF-α, ST, IL-6, TC and other variables.

### 2.2. Measurements and Methods

The routine kit method and enzymatic colorimetric method were employed for the determination of TC. The BMI levels were assessed by dividing the BW (kilograms) by the square of body height (meters) (kg/m^2^) [27,28]. The already published levels of HNW-related BMI and HOW-related BMI for the Saudi population were followed [29].

The BP (SBP and DBP) levels determined by routine methods [30] in the NT subjects were in the normal range and in HT subjects were in the HT range [31,32]. However, the subjects only with grade 1 HT in the range of 140–159/90–99 [13] were included. All BP measurements were carried out by the same person using a manual mercury sphygmomanometer (MS-S1500-Mercury Sphygmomanometer, Medical Sources Co., Limited, Nanjing, Jiangsu, China). The blood samples obtained from NC, HT and OHT groups were taken, serum-separated, and analyzed using ELISA (enzyme-linked immunosorbent assay) kits for serum Lep, TNF-α, ST, TC and IL-6 and other variables.

### 2.3. Data Analysis

The data analysis was performed using the Statistical Package for Social Sciences (version 24.0) for Windows (SPSS Inc., Chicago, IL, USA). GraphPad Prism (version 6.0) software, San Diego, CA, USA, was used. The values of the analyzed data were obtained as mean ± standard deviation (SD). The distribution of the quantitative characteristics of the present data corresponded to a normal one. Two-tailed *p*-values were obtained by applying the Student *t*-test (unpaired two-sample *t*-test). One-way analysis of variance (ANOVA) was applied to compare the variables of three subject groups. The results obtained by the Student *t*-test were further confirmed by the Tukey–Kramer post hoc test. For the determination of positive or negative correlations, linear regression lines were plotted. The values of the coefficient of determination (R^2^) were obtained, showing the level of significance. The value of significance (*p*) was obtained considering the biostatistical applications [33]. The significance level was considered as *p* ≤ 0.05.

## 3. Results

The characteristic features and related parameters in middle-aged male subjects with HT and OHT were compared (Table 1). The age of NC, HT and OHT subject groups was 55.37 ± 2.86, 55.35 ± 2.82 and 55.36 ± 2.78, respectively, having no significant difference. The HNW BMI (kg/m^2^) and HOW BMI (kg/m^2^) of these subject groups are given as a range and mean ± SD in Table 1.

### 3.1. Two-Sample Comparisons and Analysis of Variance for Serum Leptin, TNF-α, IL-6, TC, ST and Other Variables in HT and OHT Men

Comparisons for Lep, TNF-α and TC showed significant results for group comparisons (Table 1). The Lep levels in HT vs. NC (10.02 ± 6.35 vs. 5.75 ± 2.27), OHT vs. NC (13.13 ± 7.26 vs. 5.75 ± 2.27) and OHT vs. HT (13.13 ± 7.26 vs. 10.02 ± 6.35) showed highly significant variations (*p* < 0.0001). The TNF-α levels in HT vs. NC (8.25 ± 3.57 vs. 4.69 ± 2.10), OHT vs. NC (11.71 ± 5.07 vs. 4.69 ± 2.10) and OHT vs. HT (11.71 ± 5.07 vs. 8.25 ± 3.57) showed highly significant changes (*p* < 0.0001). The TC levels in HT vs. NC (189.32 ± 13.65 vs. 177.06 ± 9.49; *p* < 0.0001), OHT vs. NC (194.16 ± 12.37 vs. 177.06 ± 9.49; *p* < 0.0001) and OHT vs. HT (194.16 ± 12.37 vs. 189.32 ± 13.65; *p* < 0.01) showed significant variations.

The ST indicated a significant difference for OHT vs. NC (345.36 ± 155.80 vs. 417.96 ± 175.28; *p* < 0.002) but did not show significant variation for other comparisons. The IL-6 gave a significant difference for OHT vs. NC (10.90 ± 8.63 vs. 6.46 ± 6.23; *p* < 0.0001) and OHT vs. HT (10.90 ± 8.63 vs. 7.88 ± 6.03; *p* < 0.004) but not for HT vs. NC. The comparison for FBG and Hp did not present significant variations for HT vs. NC, OHT vs. NC, and OHT vs. HT.

One-way ANOVA presented significant change among subject groups for the levels of Lep, TNF-α, IL-6, TC and ST (Table 2). However, no significant variation was found among groups for the remaining variables.

### 3.2. Association of Serum Leptin with Other Variables in Hypertensive and Overweight-Related Hypertensive Men

The results for the association of serum Lep with other variables in NC, HT and OHT men are given in Table 3. Serum Lep plotted against TNF-α presented a highly significant positive linear correlation for the subject groups. Similar results were obtained for the association of Lep with ST, though the correlation of Lep with ST was a negative linear one. Serum Lep was associated significantly with IL-6 and TC showing a positive linear correlation for subject groups. Other variables of Hp and FBG did not present a significant correlation with Lep (Table 3).

### 3.3. Association of Serum TNF-Alpha with Other Variables in Hypertensive and Overweight-Related Hypertensive Men

Results for the association of serum TNF-alpha with other variables in the subject groups are shown in Table 3. The correlation of serum TNF-alpha with ST was found to be negatively linear. Its association with Lep, IL-6 and TC was significant with a positive linear correlation in the subject groups.

### 3.4. Association of Serum Testosterone with Other Variables in Hypertensive and Overweight-Related Hypertensive Men

The association of ST with other variables in the subject groups is shown in Table 3. The ST gave a negative linear and significant correlation with IL-6. A negative linear but non-significant correlation of ST with TC was noted in the subject groups except in the NC group, which showed a significant negative linear correlation. Lep and TNF-α were both associated with ST, showing a significant negative correlation (Table 3).

### 3.5. Association of Serum Interleukin-6 with Other Variables in Hypertensive and Overweight-Related Hypertensive Men

The association of serum IL-6 with TC, Lep and TNF-α in the subject groups (Table 3) showed positive and significant linear correlations, whereas its association with ST was negative and significant linear. Other variables did not show a significant correlation with IL-6.

### 3.6. Association of Total Cholesterol with Other Variables in Hypertensive and Overweight-Related Hypertensive Men

The association of TC with Lep, TNF-α and IL-6 (Table 3) presented significant positive linear correlations. However, non-significant correlations in the HT and OHT subject groups were obtained for TC when plotted against ST. The TC did not show significant correlations with the remaining variables.

## 4. Discussion

We attempted to assess and explore the pathophysiological role of the variations in and associations of serum Lep, TNF-α, ST, IL-6, TC, FBG and Hp in NC, HT and OHT subjects. A study on NW and OW male subjects [26] provided information on the significant variation and positive linear correlation among serum Lep, BMI and BP and various other cytokines and inflammatory factors, although that study was conducted on 18–20-year-old healthy male university students and was quite descriptive, involving various low, medium and high BMI levels in NW and OW subjects.

Considering the aim of the present study, we were more interested in exploring the changes or associations at a specific level of BMI and BP. We selected subjects at a specific BMI level of 23–24.9 kg/m^2^ (HNW BMI) for NC and HT and 28–29.9 kg/m^2^ (HOWBMI) for OHT. We could not study the LNW BMI and MNW BMI for NC and HT subjects or the LOW BMI and MOW BMI for OHT subjects. Furthermore, only grade 1 HT subjects were included in the present study. Hence, the present study is well controlled for gender, age, BMI and BP (normotensive and grade 1 HT subjects).

The values were compared for each pair of groups and among groups, and associations were found for various variables with each other. Although it would have been more interesting to obtain data from additional subjects, namely NC and HT subjects with LNW BMI and MNW BMI and OHT subjects with LOW BMI and MOW BMI, this could not be accomplished owing to insufficient funding.

We found a significant difference in serum Lep for both the HT and OHT subject groups when compared to the NC subject group. A positive correlation was found between serum Lep and OHT, and it was revealed that Lep has an important role in the pathophysiology of OHT [34]. It was found that Lep has an influencing role in OHT, and HT and endothelial dysfunction partly relate to serum levels of Lep and other adipokines [35]. Inflammation caused by cytokines causes inflammation-dependent aortic stiffening that may lead to ventricular stiffness [36]. These observations identify the impact of Lep via inflammatory processes in the progressive development of HT. Furthermore, OW and obesity can lead to the dysfunction of perivascular adipose tissue (PVAT) that releases Lep and other cytokines/chemokines to the vascular wall [37,38] and causes endothelial dysfunction and inflammation [37]. These reports support the changes found in the present study for Lep interacting with IL-6 and TNF-α in HT and OHT subjects compared to NC subjects.

The OW and obesity status increases the production of contractile factors and incorporates increased arterial vasoconstriction and greater vessel tone, producing different effects on the control of vascular tone [37]. The PVAT-derived contractile factors, e.g., Lep, cause vasoconstriction. It was found that vascular relaxation is decreased in patients with HT, which is an indirect association of Lep in HT [38].

Arterial stiffness via promoting vascular smooth muscle cell proliferation as well as migration associated with HT has been suggested to be due to Lep receptors located in vessels, especially in the aorta [39], tunica media and adventitia in arteries and in atherosclerotic plaques [40]. Furthermore, the function of Lep in promoting angiogenesis, activating the immune system, increasing platelet aggregation, producing radical oxygen species (ROS) [41] and inducing endothelial oxidative stress and ROS in experimental human cell models [42] interactively presents a mechanism whereby the risk of the development of HT increases. Adiponectin, having protective effects on arteries, is associated negatively with Lep, and hence, hypoadiponectinemia is associated with increased Lep levels [43]. Due to limited funding, adiponectin could not be determined. However, the leptin-to-adiponectin ratio will be calculated in future studies as a priority.

Another mechanism explaining our present results is that an increased activity of the sympathetic nervous system in OW subjects has been revealed in several studies [44,45,46], and it was further found that increased BW associated with HT stimulates the sympathetic stimulation causing increased development and progression of HT with an increase in Lep levels in association with an increase in pro-inflammatory cytokines, and hence a further increased HT condition [44,45,46].

The second important factor that we investigated in the present work is the increased level of TNF-α in OW and HT subjects. This investigation can be explained with the help of several related reports. The cross-linked immune system–adiposity–inflammation–blood pressure concept makes a set of vicious events [47] having a major role in HT [48]. TNF-α is an important and effective pro-inflammatory adipocytokine that is involved in causing low-grade inflammation and influencing CVDs involving OW/obesity, atherosclerosis and other disorders [49]. The OW and inflammation in HT men showed a significant role of TNF-α [23].

The low-grade inflammation related to the adipose tissue and excess fats [17] that are related to increased levels of serum TC in HT and OHT subjects in the present study is associated with immune complications leading to HT and other disorders [17,18]. There are a variety of immune factors involved in regulating the immune processes in vascular pathologies caused by the adipose-tissue-derived and most notorious pro-inflammatory cytokine TNF-α and other adipokines [50]. Probable immune-related inflammatory changes in newly diagnosed HT with higher BMI showed higher levels of TNF-α, and other factors better characterizing the CV risk in HT patients [51].

The immune mechanisms related to immune system activation explain our observations in the current clinical study. Inflammatory processes are involved in the pathogenesis and progression of CVD complications [52,53], and inflammatory markers may serve as emergent therapeutic targets [52]. Increased levels of IL-6 and TNF-α in the HT and OHT groups in the present study fit nicely in the proposed mechanism of inflammatory processes leading to CVD complications. The inflammatory status facilitated by the CV risk factors [54] is then regulated by the mechanisms related to immune system activation [51,55].

The decreased levels of ST in HT and OHT men in the present investigation, and especially the significantly decreased ST levels in OHT (OHT vs. NC), can be described with the help of other reports. The mechanism whereby testosterone decreases in response to HT in OW status has been reported, and it was found that serum levels of ST are associated with the incidence of morbidity and mortality of CVDs in men [56]. Furthermore, the effect of ST on CVD processes was revealed [57,58,59].

It has been shown in male patients with coronary heart disease (CHD) that acute intracoronary administration of ST brings coronary artery dilation and, hence, increases blood flow and improves coronary activities [57]. The long-term administration of normal ST doses decreases the symptoms of angina and cardiac ischemia in men [58,59]. The ST is lower in men with OW and obese status than in the NW people [60]. On the contrary, lifestyle changes bring ST to an increased level in men with OW and obesity [61]. The low levels of ST in men with obesity/OW increase BP, which leads to CVD complications, though it is not known how ST causes this change. OW and obesity are associated with a decrease in ST [62]. A high increase in BW is accompanied by elevated levels of aromatase from fat that decrease the hypothalamic–pituitary–testis pathway and hence cause a decrease in ST production by Leydig cells in the testes [63].

The ST deficiency that occurs in OW/obesity leads to changes in blood vessels and vice versa [20,22,64,65]. However, it has not been studied thoroughly to have a clear idea of the role of ST in OW subjects. Therefore, the association between BW change and ST variations is not simple [20]. Furthermore, there are reports showing the occurrence of hypogonadism due to decreased levels of ST associated with BW changes [22,64,65]. Hence, it seems reasonable to think that manipulations for decreasing BW can result in an increase in ST [22]. However, we clarify that besides thorough clinical and other laboratory measurements, we assayed the SHBG during diagnosis/differential diagnosis for our subjects. Based on the clinical/laboratory information and levels of SHBG, subjects with hypogonadism and other disorders were ruled out.

Subjects with increased levels of BMI were associated with the occurrence of vascular dysfunctions, decreased ST and increased inflammation [19]. This investigation is quite supportive of our observation of decreased ST and increased inflammation (IL-6 and TNF-α). Based on these observations, it was suggested that manipulating procedures leading to weight loss should be employed to increase levels of ST and decrease levels of anti-inflammatory factors that may prevent vascular dysfunction [19]. ST was found to decrease in subjects with high BMI compared to those with low BMI [19].

On the other hand, decreased levels of ST stimulate fat formation in the viscera, leading to a chain of events causing a further decrease in the levels of ST [66]. Lep levels increase in OW and obesity and have a negative effect on luteinizing hormone (LH) and the production of human chorionic gonadotropin (hCG)-stimulated testicular androgen [67]. The adipose tissue, fat formation and endocrine events of ST homeostasis and OW/obesity are associated with the inflammatory processes through the release of pro-inflammatory and anti-inflammatory factors [68] and decreased blood supply to adipocytes that causes hypoxia [69]. This then becomes a cascade causing further inflammation by releasing TNF-α and IL-6 [70,71], decreasing nitric oxide (NO) in blood vessels and causing vascular dysfunction [21]. This interactive set of investigations is a landmark in our present study.

Not only ST but also other sex hormones including estradiol have also shown cardioprotective actions in association with vascular physiology [57,59], though we could not measure the other sex hormones mainly due to a shortage of funding. Suppressing the activity of androgens was found to increase the BP in male subjects [72], which verifies the effect of ST decreasing BP in men; i.e., ST and BP are negatively associated. This indicates that any manipulation for increasing ST could result in a decrease in BP in OW and obese men [61]. Low levels of ST are associated with the morbidity and mortality of CVDs in men [61]. These reports verify our present results.

There are other studies that did not find a change in BP in response to lifestyle changes for decreasing BW [24,25]. They found little change in BW. The present study comprises NW subjects with HT and OW subjects with HT, and hence, changes occurred accordingly. Therefore, we could find a progressive increase in various variables with increased BMI in OHT compared to that in HT and NC.

Cardiovascular events in HT increase morbidity and mortality. Pharmacological interventions, exercise and diet plans including various forms of therapeutic patient education (TPE) help in reducing morbidity and mortality due to cardiovascular involvement. Such aspects have not been frequently studied considering well-organized and personalized approaches.

The data presented in the Results section clearly show the significance level for each two-group comparison (Table 1) for Lep, TNF-α, IL-6, TC and ST and among all three groups (Table 2) for various variables. Based on that, we interpreted the results with the help of already published reports. However, the results for various correlations given in Table 3 show significant associations not only for the OHT and HT groups but also for the NC group. Such results might have been due to our collection of the data of NC and HT subjects with HNW BMI and OHT subjects with HOW BMI. For further evidence, it is necessary to carry out more studies including NC and HT subjects with low and medium weight BMI and OHT subjects with low and medium overweight BMI as well. Hopefully, such management can clarify the associations of various variables with each other and with the mentioned levels of BMI.

Our research project associated with the present manuscript was accepted based on our zest and decision to further understand the reasons behind HOHT instead of LOHT and MOHT in Saudi Arabia. Indeed, it would be more interesting and helpful to include the study of the subcategories of LOW, MOW, LOHT and MOHT subjects compared to LNW, MNW, LNHT and MNHT subjects. Furthermore, although we had limited funding, our pilot studies did not show significant changes in most of the determinations in LOW, MOW, LOHT and MOHT subjects compared to LNW, MNW, LNHT and MNHT subjects. Hence, we studied the NC (NT subjects with HNW corresponding to the BMI 23–24.9 kg/m^2^), HT (HNHT corresponding to the BMI 23–24.9 kg/m^2^) and OHT (HOHT corresponding to the BMI 28–29.9 kg/m^2^) groups. BMI-related studies are carried out generally either by considering the individual values of BMI, i.e., 18, 19, 20, …, etc., or by comparing the NW-, OW- and obesity-related BMI categories. However, we have mentioned that we formed subgroups of BMI levels and studied only the HNW, HOW, HNHT and HOHT subject subgroups. We think that analyzing the data of subcategories altogether in NW or NHT and OW or OHT does not show a clear picture for a pathophysiological perspective. We suggest that studying the lower, middle and higher or upper limits of the BMI is essentially required, but studying at least the upper limits of the BMI ranges makes the data better for pathophysiological interpretation. Besides this, it was also not feasible for us to collect and analyze the data for all subcategories, mainly due to funding limitations, so we planned to carry out this study initially on HNW, HOW, HNHT and HOHT subjects.

We followed the already published data for the levels of HNW-related BMI and HOW-related BMI for the Saudi population [29]. Therefore, we suggest carrying out wide-BMI-range studies, since such studies have not been performed yet in Saudi Arabia. These suggested studies may clarify whether the correlations obtained in the present study appear in similar or different patterns in subjects with low, medium, HNW-, LNW- and HOW-related BMIs.

We mentioned the merits of collecting and analyzing the data in subcategories of high and low NW or high and low OW. It seems helpful to classify further the OW subjects into LOW, MOW and HOW. This approach is more interesting in pathophysiology since we can carry out further studies and can try to know whether the upper portion/part of the BW in OW subjects could be designated as OW or obese. Owing to the prevailing health and socioeconomic problems of the general public, there is an urgent need to investigate better standards for specifying the BMI levels of healthy NW, OW and obese people. This information would be helpful for elucidating the impact of BW variations in HT and other medical disorders.

If only the upper or high levels of BMIs (HNW-related BMI in NCs and HTs; HOW-related BMI in OHTs) show similar correlations with various variables after performing wide-BMI-range studies, then it would be necessary to suggest that the related Health Department plan to carry out comprehensive mega-studies for confirming the existing categorization/classification of BMI in Saudi population since BMI and BMI categories are region/country-based and public health/lifestyle-based specifications. It could be that the upper or high level of BMI in normal individuals is actually the low level of overweight individuals, and the upper level of the overweight BMI is actually the low level of obese individuals for the Saudi population.

Additionally, we suggest that cytokines are not only inflammatory or anti-inflammatory factors/biomarkers, but also the physiological regulatory factors/substances, as are the hormones that are also altered in physiological and pathophysiological conditions. The cytokines Lep, TNF-α and IL-6 and hormone ST in our present study are physiological regulatory factors as well. This viewpoint could partly explain the existence of significant associations/correlations between the variables to a certain extent even in the NC subject group. The comparisons in NC, HT and OHT subject groups given in Table 1 and Table 2 provide the significance levels of variations for impressive physiological/pathophysiological interpretations.

We suggest that the changes we noted in the present study would be helpful for further BMI-based studies in various subcategories of NW, OW and obese subjects with/without hypertension. We interpreted several of our results based on existing studies. However, a variety of changes at the systemic, cellular and molecular levels require investigation to consider the pathophysiological evidence of the present study.

Conclusively, the current report provides pathophysiological evidence of the interactive role of serum Lep, TNF-α, ST, TC and IL-6 in NT, HT and OHT middle-aged men. The physiological approach of assessing the biochemical parameters, including those determined in the present study, and a variety of other factors may further help in understanding the cardiovascular events and the influencing factors in HT and OHT.

## 5. Limitations

We found wide variations in our pilot studies for the age- and gender-controlled levels of serum Lep, TNF-α, IL-6, TC, ST and other variables in NC, HT and OHT men. Hence, we carried out the present study in men of a specified age—51–60 years. However, to understand the clear evidence of the general role of Lep, ST, TNF-α, inflammatory status and lipid profile in NC, HT and OHT, it seems necessary to have the data of a wide age range of both men and women. Hopefully, existing and future studies will further clarify the impact of a wide range of age in men and women.

We measured the levels of ST but could not determine the levels of other gonadal/steroid hormones and gonadotrophic hormones to understand the interplay of endocrine changes in HT and OHT.

There is a need to assess the interactive role of Lep with other adipokines, especially adiponectin, to investigate OHT. We could not include the determinations of adiponectin and other related adipokines mainly due to the limited funding available for the current report.

Indeed, other groups of subjects comprising obese and obese HT subjects are the highly important groups of subjects that could not be investigated in the present study, since our proposal was not related to obesity-related HT. However, we wish to overcome this limitation by carrying out further studies in the future for other proposals for studying obese subjects and obesity-related HT.

The data of several diseases including mainly CHD, DM, renal ischemia/failure and other kidney disorders, and cerebral ischemia/stroke were excluded in the present study though these and other diseases are prevalent in OW/obese subjects with/without HT.

## Figures and Tables

**Table 1:** Characteristic features and related parameters in normal control and hypertensive and overweight-related hypertensive middle-aged men.

Variables	Normal Weight, Hypertensive and Overweight Hypertensive Subjects								
	HT vs. NC			OHT vs. NC			OHT vs. HT		
	NC	HT	*p*-Value	NC	OHT	*p*-Value	HT	OHT	*p*-Value
Number of subjects (n)	98	97	-	98	97	-	97	97	-
Sex (male)	98	97	-	98	97	-	97	97	-
Age (years)	55.37 ± 2.86	55.35 ± 2.82	NS	55.37 ± 2.86	55.36 ± 2.78	NS	55.35 ± 2.82	55.36 ± 2.78	NS
Age range (years)	51–60	51–60	-	51–60	51–60	-	51–60	51–60	-
BMI (kg/m^2^)	24.04 ± 0.60	24.03 ± 0.61	NS	24.04 ± 0.60	29.06 ± 0.58	<0.0001	24.03 ± 0.61	29.06 ± 0.58	<0.0001
BMI range (kg/m^2^)	23–24.9	23–24.9	-	23–24.9	28–29.9	-	23–24.9	28–29.9	-
TC (mg/dL)	177.06 ± 9.49	189.32 ± 13.65	<0.0001	177.06 ± 9.49	194.16 ± 12.37	<0.0001	189.32 ± 13.65	194.16 ± 12.37	<0.01
FBG (mg/dL)	97.98 ± 6.86	97.64 ± 6.24	NS	97.98 ± 6.86	98.41 ± 7.11	NS	97.64 ± 6.24	98.41 ± 7.11	NS
IL-6 (pg/mL)	6.46 ± 6.23	7.88 ± 6.03	NS	6.46 ± 6.23	10.90 ± 8.63	<0.0001	7.88 ± 6.03	10.90 ± 8.63	<0.004
Hp (ng/mL)	11.44 ± 5.76	10.91 ± 5.49	NS	11.44 ± 5.76	11.17 ± 5.46	NS	10.91 ± 5.49	11.17 ± 5.46	NS
TNF-α (pg/mL)	4.69 ± 2.10	8.25 ± 3.57	<0.0001	4.69 ± 2.10	11.71 ± 5.07	<0.0001	8.25 ± 3.57	11.71 ± 5.07	<0.0001
ST (ng/dL)	417.96 ± 175.28	378.09 ± 179.41	NS	417.96 ± 175.28	345.36 ± 155.80	<0.002	378.09 ± 179.41	345.36 ± 155.80	NS
Lep (ng/mL)	5.75 ± 2.27	10.02 ± 6.35	<0.0001	5.75 ± 2.27	13.13 ± 7.26	<0.0001	10.02 ± 6.35	13.13 ± 7.26	<0.001

NC: normal control (normotensive with high normal weight BMI 23–24.9 kg/m^2^); HT: hypertensive (hypertensive with high normal weight BMI 23–24.9 kg/m^2^); OHT: overweight hypertensive (hypertensive with high overweight BMI 28–28.9 kg/m^2^); BMI: body mass index; TC: total cholesterol; FBG: fasting blood glucose; IL-6: interleukin-6; Hp: hepcidin; TNF-α: tumor necrosis factor alpha; ST: serum testosterone; Lep: leptin; NS: nonsignificant; values are mean ± standard deviation (SD); two-tailed *p*-value was obtained by using unpaired two-sample *t*-test.

**Table 2:** Analysis of variance for characteristic features and related parameters in normal weight, hypertensive and overweight hypertensive middle-aged men.

Variables	Normal Weight Hypertensive and Overweight Hypertensive Subjects			*p*-Value
	NC	HT	OHT	
Number of subjects (n)	98	97	97	-
Sex (male)	98	97	97	-
Age (years)	55.37 ± 2.86	55.35 ± 2.82	55.36 ± 2.78	NS
Age range (years)	51–60	51–60	51–60	-
BMI (kg/m^2^)	24.04 ± 0.60	24.03 ± 0.61	29.06 ± 0.58	<0.001
BMI range (kg/m^2^)	23–24.9	23–24.9	28–29.9	-
TC (mg/dL)	177.06 ± 9.49	189.32 ± 13.65	194.16 ± 12.37	<0.001
FBG (mg/dL)	97.98 ± 6.86	97.64 ± 6.24	98.41 ± 7.11	NS
IL-6 (pg/mL)	6.46 ± 6.23	7.88 ± 6.03	10.90 ± 8.63	<0.001
Hp (ng/mL)	11.44 ± 5.76	10.91 ± 5.49	11.17 ± 5.46	NS
TNF-α (pg/mL)	4.69 ± 2.10	8.25 ± 3.57	11.71 ± 5.07	<0.001
ST (ng/dL)	417.96 ± 175.28	378.09 ± 179.41	345.36 ± 155.80	<0.05
Lep (ng/mL)	5.75 ± 2.27	10.02 ± 6.35	13.13 ± 7.26	<0.001

NC: normal control (normotensive with high normal weight BMI 23–24.9 kg/m^2^); HT: hypertensive (hypertensive with high normal weight BMI 23–24.9 kg/m^2^); OHT: overweight hypertensive (hypertensive with high overweight BMI 23–24.9 kg/m^2^); BMI: body mass index; TC: total cholesterol; FBG: fasting blood glucose; IL-6: interleukin-6; Hp: hepcidin; TNF- α: tumor necrosis factor alpha; ST: serum testosterone; Lep: leptin; NS: nonsignificant; values are mean ± standard deviation (SD); *p*-values for one-way ANOVA (analysis of variance); statistical analysis was performed by applying the Statistical Package for Social Sciences (SPSS), version 24.0 for Windows.

**Table 3:** Association of variables in hypertensive and overweight-related hypertensive middle-aged men.

Variables	Coefficient of Determination (R^2^) and Significance Levels for Association of Variables in Normal Weight Control and Hypertensive and Overweight-Related Hypertensive Middle-Aged Men											
	Lep			TNF-α			ST			IL-6		
	NC (n: 98)	HT (n: 97)	OHT (n: 97)	NC (n: 98)	HT (n: 97)	OHT (n: 97)	NC (n: 98)	HT (n: 97)	OHT (n: 97)	NC (n: 98)	HT (n: 97)	OHT (n: 97)
TNF-α	0.62 ****	0.69 ****	0.91 ****	-	-	-	0.39 ****	0.48 ****	0.37 ****	0.38 ****	0.50 ****	0.63 ****
ST	0.34 ****	0.27 ****	0.34 ****	0.39 ****	0.48 ****	0.37 ****	-	-	-	0.11 ****	0.21 ****	0.18 ****
IL-6	0.37 ****	0.72 ****	0.73 ****	0.38 ****	0.50 ****	0.63 ****	0.11 **	0.21 ****	0.18 ****	-	-	-
Hp	0.006	0.22	0.009	0.006	0.02	0.002	0.000	0.000	0.004	0.004	0.02	0.01
TC	0.07 **	0.09 **	0.12 ***	0.12 ***	0.13 ***	0.11 ****	0.09 **	0.010	0.01	0.14 ***	0.11 ***	0.07 **
FBG	0.001	0.005	0.02	0.000	0.02	0.01	0.001	0.002	0.000	0.02	0.01	0.01
Lep	-	-	-	0.62 ****	0.69 ****	0.91 ****	0.34 ****	0.27 ****	0.34 ****	0.37 ****	0.72 ****	0.73 ****

NC: normal control (normotensive with high normal weight BMI 23–24.9 kg/m^2^); HT: hypertensive (hypertensive with high normal weight BMI 23–24.9 kg/m^2^); OHT: overweight hypertensive (hypertensive with high overweight BMI 23–24.9 kg/m^2^); BMI: body mass index; TNF-α: tumor necrosis factor alpha; ST: serum testosterone; IL-6: interleukin-6; Hp: hepcidin; TC: total cholesterol; FBG: fasting blood glucose; Lep: leptin; regression lines were plotted to obtain the values of R^2^ and the values of significance (*p*); statistical analysis was performed by applying the Statistical Package for Social Sciences (SPSS), version 24.0 for Windows; **: *p* ≤ 0.01; ***: *p* ≤ 0.001; ****: *p* ≤ 0.0001.

## Data Availability

Data are contained within the article.

## References

[1] Kearney P.M., Whelton M., Reynolds K., Muntner P., Whelton P.K., He J. (2005). Global burden of hypertension: Analysis of worldwide data. Lancet.

[2] Jaacks L.M., Vandevijvere S., Pan A., McGowan C.J., Wallace C., Imamura F., Mozaffarian D., Swinburn B., Ezzati M. (2019). The obesity transition: Stages of the global epidemic. Lancet Diabetes Endocrinol..

[3] Fantin F., Giani A., Zoico E., Rossi A.P., Mazzali G., Zamboni M. (2019). Weight Loss and Hypertension in Obese Subjects. Nutrients.

[4] Wilson P.W., D’Agostino R.B., Sullivan L., Parise H., Kannel W.B. (2002). Overweight and obesity as determinants of cardiovascular risk: The Framingham experience. Arch. Intern. Med..

[5] Linhart C., Tukana I., Lin S., Taylor R., Morrell S., Vatucawaqa P., Magliano D., Zimmet P. (2016). Continued increases in hypertension over three decades in Fiji, and the influence of obesity. J. Hypertens..

[6] Kannel W.B. (1996). Blood pressure as a cardiovascular risk factor: Prevention and treatment. JAMA.

[7] Hubert H.B., Feinleib M., McNamara P.M., Castelli W.P. (1983). Obesity as an independent risk factor for cardiovascular disease: A 26-year follow-up of participants in the Framingham Heart Study. Circulation.

[8] Zhu L., Fang Z., Jin Y., Chang W., Huang M., Chen Y., Yao Y. (2021). Circulating ERBB3 levels are inversely associated with the risk of overweight-related hypertension: A cross-sectional study. BMC Endocr. Disord..

[9] Cutler J.A., Sorlie P.D., Wolz M., Thom T., Fields L.E., Roccella E.J. (2008). Trends in hypertension prevalence, awareness, treatment, and control rates in United States adults between 1988–1994 and 1999–2004. Hypertension.

[10] Bramlage P., Pittrow D., Wittchen H.U., Kirch W., Boehler S., Lehnert H., Hoefler M., Unger T., Sharma A.M. (2004). Hypertension in overweight and obese primary care patients is highly prevalent and poorly controlled. Am. J. Hypertens..

[11] NCD Risk Factor Collaboration (NCD-RisC) (2017). Worldwide trends in blood pressure from 1975 to 2015: A pooled analysis of 1479 population-based measurement studies with 19·1 million participants. Lancet.

[12] Shihab H.M., Meoni L.A., Chu A.Y., Wang N.Y., Ford D.E., Liang K.Y., Gallo J.J., Klag M.J. (2012). Body mass index and risk of incident hypertension over the life course: The Johns Hopkins Precursors Study. Circulation.

[13] Williams B., Mancia G., Spiering W., Agabiti Rosei E., Azizi M., Burnier M., Clement D.L., Coca A., de Simone G., Dominiczak A. (2018). 2018 ESC/ESH Guidelines for the management of arterial hypertension. Eur. Heart J..

[14] Higashino R., Miyaki A., Kumagai H., Choi Y., Akazawa N., Ra S.G., Tanabe Y., Eto M., So R., Tanaka K. (2013). Effects of lifestyle modification on central blood pressure in overweight and obese men. Blood Press Monit..

[15] Ho A.K., Bartels C.M., Thorpe C.T., Pandhi N., Smith M.A., Johnson H.M. (2016). Achieving Weight Loss and Hypertension Control Among Obese Adults: A US Multidisciplinary Group Practice Observational Study. Am. J. Hypertens..

[16] Whelton P.K., Carey R.M., Aronow W.S., Casey D.E., Collins K.J., Dennison Himmelfarb C., DePalma S.M., Gidding S., Jamerson K.A., Jones D.W. (2018). 2017 ACC/AHA/AAPA/ABC/ACPM/AGS/APhA/ASH/ASPC/NMA/PCNA Guideline for the Prevention, Detection, Evaluation, and Management of High Blood Pressure in Adults: A Report of the American College of Cardiology/American Heart Association Task Force on Clinical Practice Guidelines. J. Am. Coll. Cardiol..

[17] Lechi A. (2017). The obesity paradox: Is it really a paradox? Hypertension. Eat Weight Disord..

[18] Pouvreau C., Dayre A., Butkowski E.G., de Jong B., Jelinek H.F. (2018). Inflammation and oxidative stress markers in diabetes and hypertension. J. Inflamm. Res..

[19] Aminuddin A., Salamt N., Ahmad Fuad A.F., Chin K.Y., Ugusman A., Soelaiman I.N., Wan Ngah W.Z. (2019). Vascular Dysfunction among Malaysian Men with Increased BMI: An Indication of Synergistic Effect of Free Testosterone and Inflammation. Medicina.

[20] Wu Y., Xu D., Shen H.B., Qian S.B., Qi J., Sheng X.J. (2019). The association between body mass index and testosterone deficiency in aging Chinese men with benign prostatic hyperplasia: Results from a cross-sectional study. Aging Male..

[21] Daiber A., Steven S., Weber A., Shuvaev V.V., Muzykantov V.R., Laher I., Li H., Lamas S., Münzel T. (2017). Targeting vascular (endothelial) dysfunction. Br. J. Pharmacol..

[22] Grossmann M. (2018). Hypogonadism and male obesity: Focus on unresolved questions. Clin. Endocrinol..

[23] Gonçalves C.V., Ribeiro I.S., Galantini M.P.L., Muniz I.P.R., Lima P.H.B., Santos G.S., da Silva R.A.A. (2022). Inflammaging and body composition: New insights in diabetic and hypertensive elderly men. Exp. Gerontol..

[24] Wong C.Y., Byrne N.M., O’Moore-Sullivan T., Hills A.P., Prins J.B., Marwick T.H. (2006). Effect of weight loss due to lifestyle intervention on subclinical cardiovascular dysfunction in obesity (body mass index >30 kg/m^2^). Am. J. Cardiol..

[25] Phillips C.L., Yee B.J., Trenell M.I., Magnussen J.S., Wang D., Banerjee D., Berend N., Grunstein R.R. (2009). Changes in regional adiposity and cardio-metabolic function following a weight loss program with sibutramine in obese men with obstructive sleep apnea. J. Clin. Sleep Med..

[26] Alaamri S., Serafi A.S., Hussain Z., Alrooqi M.M., Bafail M.A., Sohail S. (2023). Blood Pressure Correlates with Serum Leptin and Body Mass Index in Overweight Male Saudi Students. J. Pers. Med..

[27] Keys A., Fidanza F., Karvonen M.J., Kimura N., Taylor H.L. (1972). Indices of relative weight and obesity. J. Chronic Dis..

[28] Shah N.R., Braverman E.R. (2012). Measuring adiposity in patients: The utility of body mass index (BMI), percent body fat, and leptin. PLoS ONE.

[29] Harakeh S., Kalamegam G., Pushparaj P.N., Al-Hejin A., Alfadul S.M., Al Amri T., Barnawi S., Al Sadoun H., Mirza A.A., Azhar E. (2020). Chemokines and their association with body mass index among healthy Saudis. Saudi J. Biol. Sci..

[30] Oh S.Y., Ryue J., Hsieh C.H., Bell D.E. (1991). Eggs enriched in omega-3 fatty acids and alterations in lipid concentrations in plasma and lipoproteins and in blood pressure. Am. J. Clin. Nutr..

[31] Basile J.N. (2008). Rationale for fixed-dose combination therapy to reach lower blood pressure goals. South Med. J..

[32] de Faria A.P., Modolo R., Fontana V., Moreno H. (2014). Adipokines: Novel players in resistant hypertension. J. Clin. Hypertens..

[33] Zahir H., Javaid A., Rehman R., Hussain Z. (2014). Statistical concepts in biology and health sciences. J. Ayub. Med. Coll. Abbottabad..

[34] Seven E. (2015). Overweight, hypertension and cardiovascular disease: Focus on adipocytokines, insulin, weight changes and natriuretic peptides. Dan. Med. J..

[35] Zachariah J.P., Hwang S., Hamburg N.M., Benjamin E.J., Larson M.G., Levy D., Vita J.A., Sullivan L.M., Mitchell G.F., Vasan R.S. (2016). Circulating Adipokines and Vascular Function: Cross-Sectional Associations in a Community-Based Cohort. Hypertension.

[36] Zanoli L., Di Pino A., Terranova V., Di Marca S., Pisano M., Di Quattro R., Ferrara V., Scicali R., Rabuazzo A.M., Fatuzzo P. (2018). Inflammation and ventricular-vascular coupling in hypertensive patients with metabolic syndrome. Nutr. Metab. Cardiovasc. Dis..

[37] Fernández-Alfonso M.S., Gil-Ortega M., García-Prieto C.F., Aranguez I., Ruiz-Gayo M., Somoza B. (2013). Mechanisms of perivascular adipose tissue dysfunction in obesity. Int. J. Endocrinol..

[38] Szasz T., Bomfim G.F., Webb R.C. (2013). The influence of perivascular adipose tissue on vascular homeostasis. Vasc. Health Risk Manag..

[39] Purdham D.M., Zou M.X., Rajapurohitam V., Karmazyn M. (2004). Rat heart is a site of leptin production and action. Am. J. Physiol. Heart Circ. Physiol..

[40] Parhami F., Tintut Y., Ballard A., Fogelman A.M., Demer L.L. (2001). Leptin enhances the calcification of vascular cells: Artery wall as a target of leptin. Circ. Res..

[41] Werner N., Nickenig G. (2004). From fat fighter to risk factor: The zigzag trek of leptin. Arterioscler. Thromb. Vasc. Biol..

[42] Yamagishi S.I., Edelstein D., Du X.L., Kaneda Y., Guzmán M., Brownlee M. (2001). Leptin induces mitochondrial superoxide production and monocyte chemoattractant protein-1 expression in aortic endothelial cells by increasing fatty acid oxidation via protein kinase A. J. Biol. Chem..

[43] Catharina A.S., Modolo R., Ritter A., Sabbatini A., Correa N., Brunelli V., Fraccaro N., Almeida A., Lopes H., Moreno H. (2017). [PP.05.34] metabolic syndrome-related features in controlled and resistant hypertensive subjects. J. Hypertens..

[44] Grassi G., Seravalle G., Brambilla G., Buzzi S., Volpe M., Cesana F., Dell’oro R., Mancia G. (2014). Regional differences in sympathetic activation in lean and obese normotensive individuals with obstructive sleep apnoea. J. Hypertens..

[45] Grassi G., Mark A., Esler M. (2015). The sympathetic nervous system alterations in human hypertension. Circ. Res..

[46] Grassi G., Pisano A., Bolignano D., Seravalle G., D’Arrigo G., Quarti-Trevano F., Mallamaci F., Zoccali C., Mancia G. (2018). Sympathetic Nerve Traffic Activation in Essential Hypertension and Its Correlates: Systematic Reviews and Meta-Analyses. Hypertension.

[47] Kaur J., Mukheja S., Varma S., Kalra H.S., Khosa B.S., Vohra K. (2020). Serum progranulin/tumor necrosis factor-α ratio as independent predictor of systolic blood pressure in overweight hypertensive patients: A cross-sectional study. Egypt Heart J..

[48] Virdis A., Dell’Agnello U., Taddei S. (2014). Impact of inflammation on vascular disease in hypertension. Maturitas.

[49] Maachi M., Piéroni L., Bruckert E., Jardel C., Fellahi S., Hainque B., Capeau J., Bastard J.P. (2004). Systemic low-grade inflammation is related to both circulating and adipose tissue TNFalpha, leptin and IL-6 levels in obese women. Int. J. Obes. Relat. Metab. Disord..

[50] Aladhami A.K., Unger C.A., Ennis S.L., Altomare D., Ji H., Hope M.C., Velázquez K.T., Enos R.T. (2021). Macrophage tumor necrosis factor-alpha deletion does not protect against obesity-associated metabolic dysfunction. FASEB J..

[51] Perticone M., Zito R., Miceli S., Pinto A., Suraci E., Greco M., Gigliotti S., Hribal M.L., Corrao S., Sesti G. (2019). Immunity, Inflammation and Heart Failure: Their Role on Cardiac Function and Iron Status. Front. Immunol..

[52] Raggi P., Genest J., Giles J.T., Rayner K.J., Dwivedi G., Beanlands R.S., Gupta M. (2018). Role of inflammation in the pathogenesis of atherosclerosis and therapeutic interventions. Atherosclerosis.

[53] Ruparelia N., Choudhury R. (2020). Inflammation and atherosclerosis: What is on the horizon?. Heart.

[54] Tedgui A., Mallat Z. (2006). Cytokines in atherosclerosis: Pathogenic and regulatory pathways. Physiol. Rev..

[55] Wolf D., Ley K. (2019). Immunity and Inflammation in Atherosclerosis. Circ. Res..

[56] Corona G., Rastrelli G., Monami M., Guay A., Buvat J., Sforza A., Forti G., Mannucci E., Maggi M. (2011). Hypogonadism as a risk factor for cardiovascular mortality in men: A meta-analytic study. Eur. J. Endocrinol..

[57] Webb C.M., McNeill J.G., Hayward C.S., de Zeigler D., Collins P. (1999). Effects of testosterone on coronary vasomotor regulation in men with coronary heart disease. Circulation.

[58] English K.M., Steeds R.P., Jones T.H., Diver M.J., Channer K.S. (2000). Low-dose transdermal testosterone therapy improves angina threshold in men with chronic stable angina: A randomized, double-blind, placebo-controlled study. Circulation.

[59] Mathur A., Malkin C., Saeed B., Muthusamy R., Jones T.H., Channer K. (2009). Long-term benefits of testosterone replacement therapy on angina threshold and atheroma in men. Eur. J. Endocrinol..

[60] Foresta C., Di Mambro A., Pagano C., Garolla A., Vettor R., Ferlin A. (2009). Insulin-like factor 3 as a marker of testicular function in obese men. Clin. Endocrinol..

[61] Kumagai H., Miyaki A., Higashino R., Akazawa N., Choi Y. (2014). Lifestyle modification-induced increase in serum testosterone and SHBG decreases arterial stiffness in overweight and obese men. Artery Res..

[62] Kelly D.M., Jones T.H. (2015). Testosterone and obesity. Obes. Rev..

[63] Dandona P., Dhindsa S. (2011). Update: Hypogonadotropic hypogonadism in type 2 diabetes and obesity. J. Clin. Endocrinol. Metab..

[64] Lamm S., Chidakel A., Bansal R. (2016). Obesity and hypogonadism. Urol. Clin. N. Am..

[65] Mulhall J.P., Trost L.W., Brannigan R.E., Kurtz E.G., Redmon J.B., Chiles K.A., Lightner D.J., Miner M.M., Murad M.H., Nelson C.J. (2018). Evaluation and Management of Testosterone Deficiency: AUA Guideline. J. Urol..

[66] Hamilton E.J., Gianatti E., Strauss B.J., Wentworth J., Lim-Joon D., Bolton D., Zajac J.D., Grossmann M. (2011). Increase in visceral and subcutaneous abdominal fat in men with prostate cancer treated with androgen deprivation therapy. Clin. Endocrinol..

[67] Isidori A.M., Caprio M., Strollo F., Moretti C., Frajese G., Isidori A., Fabbri A. (1999). Leptin and androgens in male obesity: Evidence for leptin contribution to reduced androgen levels. J. Clin. Endocrinol. Metab..

[68] Lafontan M. (2005). Fat cells: Afferent and efferent messages define new approaches to treat obesity. Annu. Rev. Pharmacol. Toxicol..

[69] Cinti S., Mitchell G., Barbatelli G., Murano I., Ceresi E., Faloia E., Wang S., Fortier M., Greenberg A.S., Obin M.S. (2005). Adipocyte death defines macrophage localization and function in adipose tissue of obese mice and humans. J. Lipid Res..

[70] Karastergiou K., Mohamed-Ali V. (2010). The autocrine and paracrine roles of adipokines. Mol. Cell. Endocrinol..

[71] Ellulu M.S., Khaza’ai H., Abed Y., Rahmat A., Ismail P., Ranneh Y. (2015). Role of fish oil in human health and possible mechanism to reduce the inflammation. Inflammopharmacology.

[72] Smith J.C., Bennett S., Evans L.M., Kynaston H.G., Parmar M., Mason M.D., Cockcroft J.R., Scanlon M.F., Davies J.S. (2001). The effects of induced hypogonadism on arterial stiffness, body composition, and metabolic parameters in males with prostate cancer. J. Clin. Endocrinol. Metab..

